# Vitamin D and COVID-19: Clinical Evidence and Immunological Insights

**DOI:** 10.3390/life15050733

**Published:** 2025-04-30

**Authors:** Olga Adriana Caliman-Sturdza, Roxana Elena Gheorghita, Iuliana Soldanescu

**Affiliations:** 1 Faculty of Medicine and Biological Sciences, Stefan cel Mare University of Suceava, 720229 Suceava, Romania; olga.caliman-sturdza@usm.ro; 2 Suceava Emergency County Clinical Hospital, 720224 Suceava, Romania; 3 Integrated Center for Research, Development, and Innovation for Advanced Materials, Nanotechnologies, Manufacturing and Control Distributed Systems (MANSiD), Stefan cel Mare University of Suceava, 720229 Suceava, Romania; iuliana.soldanescu@usm.ro

**Keywords:** SARS-CoV-2, immune mechanisms, COVID-19 outcomes, long COVID

## Abstract

Vitamin D has emerged as a potential modulator of immune responses, sparking interest in its role in COVID-19 susceptibility and clinical outcomes. This review synthesizes current clinical evidence and explores immunological insights into the relationship between vitamin D levels and COVID-19 infection severity. Epidemiological studies indicate an inverse correlation between vitamin D deficiency and an increased risk of severe disease, hospitalization, and mortality in COVID-19 patients. Immunologically, vitamin D exerts regulatory effects on both innate and adaptive immunity, enhancing antimicrobial defense mechanisms, reducing excessive inflammatory responses, and potentially mitigating cytokine storm events observed in severe COVID-19 cases. Despite promising observational data, clinical trials evaluating vitamin D supplementation have shown mixed results, underscoring the need for standardized dosing regimens and patient stratification. Future research should focus on large-scale randomized controlled trials to conclusively determine the therapeutic potential and optimal supplementation strategies for vitamin D in managing COVID-19.

## 1. Introduction

The research community has intensely studied vitamin D as a potential element to be used to manage COVID-19. The immune system is strongly supported by vitamin D, which affects both innate and adaptive immunological responses [1]. This substance can manage inflammatory responses and cytokine reactions, which in turn could reduce the severity of inflammatory conditions, like the cytokine storm observed in severe COVID-19 [2,3]. Before COVID-19, researchers observed that vitamin D deficiency elevated patient susceptibility to infections of the respiratory system, which prompted assessments of vitamin D’s effects on COVID-19 outcomes [4]. Research has shown that poor vitamin D concentration leads to enhanced possibilities of experiencing severe COVID-19 complications that result in hospitalization, intensive care requirements, and even death [5,6]. The results from clinical trials that tested vitamin D supplements have shown inconsistent findings. The data show that these supplements might provide advantages when the baseline vitamin D levels remain low [7]. Other research indicates that vitamin D supplementation has no substantial effects on disease prevention or decreased disease severity after infection [8,9]. General health organizations recommend testing vitamin D levels to check whether they are >30 ng/mL or 75 nmol/L because this ensures proper health conditions, especially for the elderly, as well as those with darker skin, those who receive minimal sun exposure, and those experiencing winter months or pandemic situations [10]. Scientists agree that vitamin D supplements are generally safe for most adults who take recommended doses between 800 and 2000 IU/day, and they should use these supplements instead of other preventive measures, such as vaccination and social distancing, along with hygiene practices and wearing masks [11]. Developing adequate vitamin D levels is useful in terms of both immune health and reducing COVID-19 severity risks, especially for people who have vitamin D deficiency [12,13,14]. Further research is required to establish whether vitamin D supplements affect COVID-19 prevention or treatment results. This narrative review attempts to summarize the current knowledge regarding the role of vitamin D in the immune mechanisms that are involved in SARS-CoV-2 infection, as well as its role in the management of COVID-19. This study also analyzes the current evidence regarding the involvement of vitamin D in the evolution of SARS-CoV-2 infection towards severe forms and complications. Finally, this review attempts to provide data on the action of vitamin D on the symptoms of long COVID and its role in the evolution of patients with long COVID.

## 2. Methods

The PubMed (National Library of Medicine, U.S. Department of Health and Human Services) and Google Scholar (Mountain View, CA, USA) databases were searched for this review from December 2020 to March 2025 for articles using the following terms: COVID-19, SARS-CoV2, vitamin D, vitamin D supplements, COVID-19 immune mechanisms, COVID clinical trials, UTI, COVID-19 severe, and long COVID. Subsequently, the references of the articles were also searched, and we tried to identify other relevant articles that were omitted during the initial search, but there were not many found. Original studies, systematic reviews, and narratives that studied the effects of vitamin D on SARS-CoV-2 infection were included in the search and we found 145 representative articles. Articles published in languages other than English were excluded, as well as articles that were preprints (such as arXiv and medRxiv), letters to the editor, commentaries, case reports, and articles without full-text content. We preferentially selected articles from journals located in the Q1-Q2 quartile. This review attempts to provide a synthesis of meta-analyses and narrative reviews, research articles, and clinical trials on the role of vitamin D in SARS-CoV-2 infection, to help researchers understand the importance of correcting vitamin D deficiency in the prevention and progression of COVID-19.

## 3. Immune Mechanisms of Vitamin D in Viral Infections

**Figure 1:**
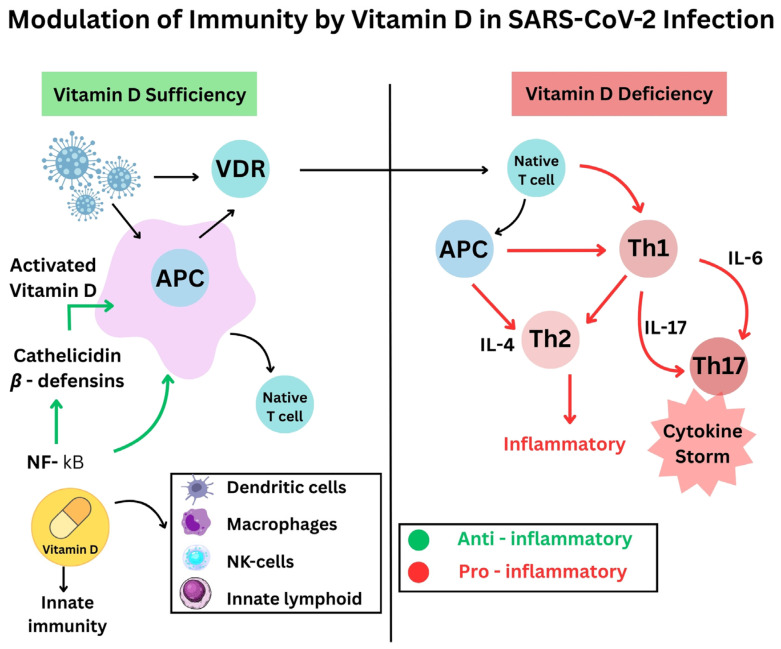
Differential effects of vitamin D sufficiency and deficiency on the immune response in the context of SARS-CoV-2 infection. The left side highlights the role of vitamin D in supporting immunity through activation of the VDR receptor, inhibition of the NF-κB pathway, and induction of antimicrobial peptide synthesis (cathelicidins, β-defensins), contributing to a balanced anti-inflammatory immune response. The right side outlines the consequences of vitamin D deficiency, which favors the activation of Th1 and Th17 cells, with excessive production of proinflammatory cytokines (IL-6, IL-17), which may lead to the cytokine storm.

**Figure 2:**
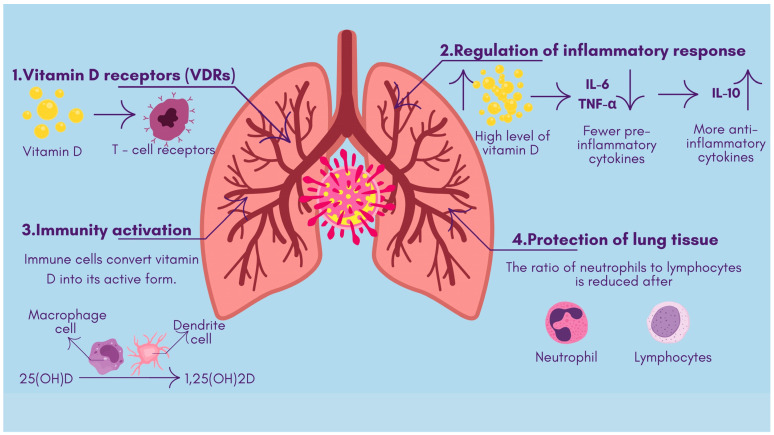
The mechanisms of action of vitamin D in viral infections. 1. The interaction between vitamin D and T-cell receptors. 2. A high level of vitamin D reduces the production of pro-inflammatory cytokines and stimulates the production of anti-inflammatory cytokines. 3. The process by which immune cells, such as macrophages and dendritic cells, transform the inactive form of vitamin D into the active form. 4. Vitamin D treatment reduces the ratio of neutrophils to lymphocytes, indicating an improvement in the state of the immune system (↑—increased value, ↓—decreased value).

The immune modulatory function of vitamin D affects both natural and adaptive immune system responses. Researchers have identified several pathways through which proper vitamin D levels aid in combating viral infection and providing severe inflammation protection against SARS-CoV-2 viruses:

✓ The antimicrobial effects of vitamin D include both viral replication suppression and protection of respiratory tract epithelial barriers [15]. Antimicrobial peptides, including defensins and cathelicidin, develop from vitamin D activation, improve lung mucosal protection, and directly destroy viruses [16].
✓ Sufficiently high vitamin D levels tend to inhibit overactive inflammatory responses in bodily systems. The use of vitamin D alters T-cell inflammation by turning dangerous pro-inflammatory Th1 and Th17 cells into safer anti-inflammatory Th2 and Treg cell responses [17]. When vitamin D levels rise, the immune system releases fewer pro-inflammatory cytokines, like IL-6 and TNF-α, while increasing the production of anti-inflammatory cytokines, such as IL-10, thus stopping the hyperactive “cytokine storm”. Active vitamin D modifies immune response control mechanisms to stabilize inflammatory processes, thereby preventing dangerous cytokine storm development [18] (Figure 1).
✓ The immune system cells, like macrophages and dendritic cells, that possess a vitamin D receptor undertake the local transformation of 25(OH)D to active 1,25(OH)_2D. Such local activity enables vitamin D to enhance innate immune responses [19]. The signaling mechanisms of vitamin D help macrophages fight pathogens while simultaneously controlling their inflammatory release. Research demonstrates that vitamin D stimulates both regulatory T-cell population magnitude and activity, which helps regulate immune system reactions [20].
✓ When vitamin D controls natural immune activation and helps to resolve inflammatory reactions, it diminishes the level of lung tissue damage. Patients with COVID-19 develop severe illness when their bodies display elevated neutrophil-to-lymphocyte ratios combined with excessive inflammation. A pilot study revealed that patients given vitamin D treatment (calcifediol) had an increased number of lymphocytes and reduced neutrophil-to-lymphocyte ratio values, which showed that they had better immune markers of severe COVID-19 [21]. The immunomodulatory properties of vitamin D potentially allow for milder inflammatory damage to the body while fighting infection (Figure 2).

## 4. Clinical Trials on Vitamin D and COVID-19 Outcomes

Medical studies have examined whether vitamin D supplements prevent COVID-19 contamination in patients and enhance their therapeutic results after becoming infected. A total of 6200 adults participated in the UK CORONAVIT Trial (2022) through its phase 3 “test-and-treat” design, in which vitamin D levels were measured so that supplements could be given to vitamin D-deficient patients for six months [22]. According to the research findings, vitamin D supplementation did not minimize either the number of COVID-19 infections or the incidence of respiratory ailments in participants or control subjects [22]. This extensive population-based pragmatic study proved that vitamin D correction in the general public did not reduce the number of COVID-19 infections or disease severity in normal community settings. The double-blind randomized controlled trial (RCT) by Murai et al. in JAMA in 2021 (n = 240 hospitalized patients) provided one large dose of 200,000 IU of vitamin D3 to patients who had moderate-to-severe COVID-19 [23]. The results showed no benefits; the median hospital stay was 7 days for both the vitamin D and placebo groups; and the differences in ICU admission, the need for ventilation, and in-hospital mortality (around 8% absolute differences) were not statistically significant. The one-time large-dose vitamin D given to sick patients in hospitals showed no positive results according to the study findings. Research from JAMA indicates that when also considering previous trials of respiratory conditions, such high-dose vitamin D supplementation should not become a routine treatment option for COVID-19 hospital patients [23]. Castillo et al. (2020) [24] conducted a pilot study in Córdoba, Spain, in which they monitored the effects of calcifediol (25-hydroxyvitamin D)-supplemented care on hospitalized patients (n = 76). The calcifediol group had a dramatically lower rate of ICU admission (only 1 of 50 patients) compared to the control group (13 of 26 patients), a difference corresponding to an odds ratio of 0.02 for ICU transfer [24]. This suggests a possible significant benefit of rapid vitamin D correction, but the trial’s design has been debated because it is a pilot study with a small sample size and some methodological limitations, so the results should be interpreted with caution [25]. A clinical study on Indian patients with mild COVID-19 symptoms or no symptoms who also had vitamin D deficiency received 60,000 IU daily for 7 days (2020, “SHADE” trial, n = 40 outpatients) [26]. The participants in the vitamin D regimen obtained high 25(OH)D levels above 50 ng/mL, whereas only 20.8% in the placebo group remained virus-positive after 21 days (*p* = 0.018) [26]. Research evidence revealed how vitamin D supplementation caused the level of fibrinogen markers to decrease in treated patients. Evidence from this small study shows that significant vitamin D supplementation may accelerate the elimination of the virus in patients with mild COVID-19 infection, but more extensive research is needed to validate these findings. The COVIT-TRIAL study in France tested the effects of vitamin D supplementation on elderly inpatients when comparing single high doses (400,000 IU) to lower doses (50,000 IU) upon admission [27]. This research revealed that patients in the high-dose group demonstrated reduced mortality compared to the patients in the low-dose group at day 14, but the mortality rates became comparable by day 28 [27]. The findings from this research indicate that critically ill elderly patients receiving a high bolus injection of vitamin D might have shorter-term protection, but this benefit did not continue past one month of treatment. One clinical evaluation at a Mexican research site (2020, n = 198 healthcare workers) demonstrated that a daily vitamin D intake of 4000 IU for 30 days lowered SARS-CoV-2 infection risk by 77% compared to placebo distribution [28]. Testing a single 500,000 IU administrated dose on 218 hospitalized patients in Argentina (2021) showed that there were no changes in respiratory measures and mortality statistics compared to the placebo group [29]. Research conducted in Saudi Arabia (2022) analyzed the recovery rate of hospitalized patients (n = 69) between 1000 IU daily and 5000 IU daily supplementation and found quicker symptom resolution primarily for cough and diminished taste without differences in disease length or fever duration [30].

The 2022 study published by Gibbons in Scientific Reports examined the impact of vitamin D supplementation on COVID-19 outcomes among U.S. veterans [10]. Vitamin D2 and D3 supplementation were associated with a 28% and 20% reduction in COVID-19 infection risk, respectively. Vitamin D3 supplementation was correlated with a 33% decrease in 30-day mortality following COVID-19 infection. While vitamin D2 showed a 25% reduction, this was not statistically significant. Higher cumulative and daily doses of vitamin D3 were linked to greater reductions in infection risk, especially among individuals with baseline serum vitamin D levels below 20 ng/mL. Black veterans experienced a more substantial reduction in infection risk from vitamin D3 supplementation compared to white veterans, suggesting potential benefits in addressing racial disparities in COVID-19 outcomes. This study suggests that vitamin D3 supplementation could serve as a safe, affordable, and accessible strategy to mitigate COVID-19 infection and mortality risks, particularly in populations with vitamin D deficiency. However, the authors emphasize the need for randomized controlled trials to establish a causal relationship and inform public health recommendations. The outcomes of clinical trials have presented conflicting results so far. The combination of research conducted with vitamin D-deficient study groups or those using calcifediol indicates that vitamin D supplementation shows potential for reducing ICU admissions and accelerating recovery paths [22,25,31,32]. Large-scale trials show that vitamin D supplementation with high doses does not improve the main clinical indicators, such as hospital stay duration, patient mortality rates, and microbial protection outcomes among hospitalized patients [21,22,33]. The diverse research results arise from small sample sizes and different vitamin D administration methods and patient demographic groups. The available evidence from these trials shows that vitamin D remains safe for use in such cases but does not support its effectiveness as a sole or preventive measure against COVID-19 infection [33,34]. Additional large-scale randomized controlled trials with high technical standards must be performed to establish vitamin D’s therapeutic role. There are one hundred and nineteen studies registered on clinicaltrials.gov regarding COVID-19 and vitamin D: five of them are in the recruitment period, seventy-six are complete, six have ended, twenty-three have an unknown status, and five have been withdrawn.

The table below (Table 1) compiles all known clinical studies (interventional trials and observational studies) investigating the role of vitamin D in COVID-19 and registered on ClinicalTrials.gov (NCT numbers) and the EU Clinical Trials Register (EudraCT numbers). Both interventional and observational studies are included, with the study type indicated, as well as studies from all countries and with all recruitment statuses (ongoing, completed, etc.). Recruitment status refers to the most updated status obtained from the registry, and Results Posted indicates whether the results have been posted on the registry. Each study entry includes a direct link to its registry record. Current data suggest that low vitamin D levels are associated with an increased risk of COVID-19 infection, as well as more complications during infection. However, studies looking at the role of vitamin D in SARS-CoV-2 infection do not provide sufficient evidence to justify its use in the management and prevention of COVID-19 alongside other established therapies.

A meta-analysis performed by Zhang et al. examined sixteen research studies (comprising eight RCTs with eight cohort studies) among 3359 patients to assess the effects of vitamin D supplementation on COVID-19 outcomes [76]. Vitamin D supplementation failed to produce a meaningful decrease in death rates among patients and their risk of hospital admission in intensive care units or the need for mechanical ventilation. This study’s conclusion requires careful interpretation because the research quality remains low and the number of participants small, and the control factors varied regarding the doses administered to patients across diverse study designs. The available data, obtained through both this recent meta-analysis and previous studies, provide insufficient evidence to determine the impact of vitamin D supplementation on immune function and cytokine storm prevention. Research indicated that higher vitamin D amounts might decrease infection frequency, yet no substantial evidence has demonstrated its benefits in terms of clinical mortality rates and intensive care unit admissions. The researchers incorporated better methodologies and adjusted odds ratios to minimize bias, which made the study results more dependable than those of prior analyses. The complete strength of the overall findings was limited due to a lack of demographic information and differences across the studied papers.

## 5. Dosage Recommendations and Guidelines

Modern healthcare experts endorse sufficient vitamin D intake for supporting complete health along with immune system function. Adults following standard vitamin D intake recommendations should take 15 to 20 micrograms per day, which equals 600 to 800 IU [77]. The government of the United Kingdom suggests taking 10 µg (400 IU) of supplements daily due to sunlight scarcity in winter and fall [78]. In all situations, 400 IU of supplements should be consumed daily by those who face substantial vitamin D deficiency risks, such as the elderly in care facilities, along with dark-skinned individuals and young children [79]. The healthcare objective focuses on sustaining 25(OH)D serum concentrations above 20–30 ng/mL because researchers hope that this range is sufficient to promote bone health and a stronger immune response [80]. Some COVID-19 guidance specifically focuses on delivering vitamin D supplements to elderly patients, individuals with obesity, and those having chronic illnesses who commonly fall within high-risk categories [81]. The UK government distributed vitamin D supplements free of charge at 400 IU per day across 2.5+ million clinically vulnerable people for 4 months due to maintaining sufficient levels as per research. Medical professionals support the need for high-risk people to receive additional treatment through daily doses between 1000 and 2000 IU and loading doses to reduce deficiency quickly, but individual requirements must guide these treatments. Official healthcare organizations emphasize deficiency correction as an essential part of overall healthcare. Although the authorities have not directly supported the use of high-dose vitamin D for COVID-19 prevention, they maintain that the official recommended dosages protect against COVID-19 for vulnerable populations [82]. Different institutions have failed to establish a minimum effective dosage level for treating COVID-19 patients. A clinical trial implementation of vitamin D included daily doses of 5000 IU alongside a single administration of 200,000–500,000 IU. Medical facilities feed COVID-19 patients who lack sufficient vitamin D with large single-dose infusions, seeking rapid level enhancement. Health organizations avoid recommending routine high-dose vitamin D treatment for hospitalized patients because of inconsistent trial results [83]. The clinical trial NCT04334005 tested the administration of a single dose of 25,000 IU of vitamin D as an immunomodulatory agent that would improve the prognosis in non-severe COVID-19 patients as a supplement in addition to standard therapy [84]. Another study (NCT04363840) recommended the administration of a dose of 50,000 IU of vitamin D once a week for 2 weeks, in combination with 81 mg of acetylsalicylic acid, but this study was withdrawn due to a lack of funding [48,84] The standards of care require clinicians to undertake vitamin D supplementation using protocols for deficiency treatment, which might include 50,000 IU doses weekly for several weeks, though this type of care does not prove effective against COVID-19 [85]. Research organizations maintain that vitamin D remains vital for general health; however, studies fail to establish it as an effective COVID-19 treatment. Data currently lack the necessary support to be used to determine whether vitamin D works for preventing or treating COVID-19 according to the U.S. NIH COVID-19 Treatment Guidelines [83]. The UK’s NICE rapid guidelines (2020) indicated that research lacks clear evidence for vitamin D usage in COVID-19 treatment yet recommended continuing standard recommendations for daily 10 µg supplementation for bone health (which provides possible indirect educational benefits for respiratory infections) [83]. Government health agencies at the NHS level declare that insufficient evidence exists that supports taking vitamin D solely to handle or protect against COVID-19 cases [86]. People should take vitamin D according to public recommendations, which state its use for health promotion and deficiency prevention. High-dose vitamin D consumption must be monitored through medical supervision whenever it exceeds suggested daily guidelines. Vitamin D accumulates in fat tissue, which means that users need to watch their intake for safety reasons. Medical experts have established that daily, adults can tolerate upper intake levels of 100 µg (4000 IU) of vitamin D supplements [87]. The established threshold for vitamin D safety applies to most individuals in the general population. The use of the very high vitamin D doses that researchers administered in trials to assess effectiveness represents a clinical study methodology but should not be conducted as regular practice because this can lead to safety issues when patients are unsupported [87,88,89,90]. Taking too much vitamin D can cause elevated calcium levels in the blood, which may result in kidney stones, confusion, and heart arrhythmias [91,92,93]. The official COVID-19 recommendations emphasize maintaining sufficient vitamin D levels through moderate daily supplementation unless a person needs clinical attention for vitamin D deficiency or participates in active clinical trials [83] (Table 2).

## 6. Vitamin D Status and Severe COVID-19 Outcomes

Research on COVID-19 severity in relation to vitamin D levels continues to grow in quantity in the scientific literature. Most observational studies suggest a link between vitamin D deficiency and adverse COVID-19 outcomes, but such findings do not establish causality [83,87]. Research shows that COVID-19 progresses more severely when patients have lower vitamin D levels when compared to people with milder illness status and the average population [89]. Vitamin D deficiency prevalence was higher among 335 COVID-19 patients than 560 healthy controls as researchers recorded 10.6 ng/mL median 25(OH) D in COVID-19 patients and 13 ng/mL in controls [94]. People with deficiency displayed greater disease severity according to study findings. A review of 17 studies that examined 2756 COVID-19 patients demonstrated that vitamin D-deficient patients faced significantly increased risks of poor disease outcomes with higher death rates, longer hospital stays, and higher admission frequencies [95]. Several examinations of COVID-19 patients have established that those who are vitamin D-deficient or less replete than recommended experience more serious health consequences along with higher fatality rates [96,97,98]. Several studies analyzing previous data show an association between vitamin D levels in patients and their need for intensive care admission and survival chances [99,100]. Sartini et al. conducted a systematic review and meta-analysis to determine whether preventive vitamin D supplementation reduces the risk of COVID-19 infection and related adverse outcomes (intensive care unit admission and mortality) [7]. Preventive vitamin D supplementation was associated with a significantly lower risk of COVID-19 infection. In RCTs, vitamin D supplementation reduced the odds of contracting COVID-19 by around 60% (pooled OR ≈ 0.40, 95% CI 0.22–0.75). Similarly, observational studies showed roughly 40% lower odds of infection in vitamin D-supplemented groups (OR ≈ 0.59, 95% CI 0.48–0.74) despite high between-study heterogeneity (I^2^ ~99%) [101,102,103,104]. Vitamin D supplementation was also associated with a markedly reduced likelihood of ICU admission among those who did become infected (pooled OR ≈ 0.32, 95% CI 0.15–0.68) [14,89,105]. However, no significant reduction in COVID-19 mortality was observed in the pooled observational data (OR ≈ 0.88, 95% CI 0.67–1.17), although one small RCT reported a significant mortality benefit (OR ≈ 0.16, 95% CI 0.03–0.83 [10,14,89,106,107,108]. In conclusion, preventive vitamin D supplementation was associated with a significant decrease in the risk of SARS-CoV-2 infection and the need for ICU-level care. These findings support the protective role of vitamin D in COVID-19 prevention; however, evidence of a mortality benefit remains inconclusive, thus warranting further investigation. Nursing home patients who consumed regular high-dose vitamin D supplements had increased survival rates and milder COVID-19 according to research in France [109]. The same result was documented in a UK study involving 444 patients with a median age of 74 [110]. Observational evidence shows that sufficient vitamin D status before COVID-19 diagnosis seems to be associated with better outcomes.

The application of vitamin D supplements within intervention-based trials resulted in a 65% reduction in relative ICU admissions and an approximately 54% decreased mortality risk for COVID-19 patients [111]. This analysis includes different study types showing protective results, although the evidence base comes mainly from observational studies rather than randomized trials. Available research evidence does not consistently demonstrate that vitamin D influences COVID-19 severity levels. It is possible that low levels of vitamin D are reflective of different risk factors [106]. Another important consideration is that low levels of vitamin D may simply be a marker of other risk factors, such as older age, obesity, and chronic illness, all of which can lead to both reduced vitamin D levels and poorer COVID-19 outcomes [100,106,112,113]. An evaluation of vitamin D levels in 348,598 UK Biobank participants who acquired COVID-19 (449 cases) revealed that factors such as age, obesity, ethnicity, and sex did not result in vitamin D levels being linked to COVID-19 infection probability [110]. Likewise, multiple meta-analyses failed to establish a significant relationship between low baseline vitamin D values (<20 ng/mL) and critical COVID-19 results [112,114,115,116,117]. This research indicates that vitamin D deficiency alone may not create serious COVID-19 conditions, while other high-risk medical situations are more likely to cause severe illness paths. Disease severity, along with illness, triggers a decrease in vitamin D levels, thus creating interpretive confusion. The data show that acute inflammation reduces the concentration of 25(OH) D in serum based on studies that demonstrated a swift decrease in vitamin D levels following inflammatory challenges [114,118]. The intense inflammatory response in COVID-19 patients can lower vitamin D levels; thus, the viral infection itself might cause vitamin D insufficiency instead of poor vitamin D status acting as a risk factor for severe disease [119]. This reverse causality effect is a recognized issue because critically ill patients generally exhibit low vitamin D levels, yet late-stage vitamin D supplementation could possibly fail to improve clinical results. Various studies demonstrate that vitamin D deficiency presents frequently in patients with severe COVID-19, yet research shows that vitamin D deficiency predisposes subjects to hospitalization or death from this disease [120,121,122]. The data show enough evidence for researchers to develop a hypothesis about how to achieve better results through enhanced vitamin D status. The associations between severe COVID-19 disease and vitamin D deficiency cannot be used to prove causation because confounders exist, and the disease itself could reduce vitamin D levels. In the medical context, it makes sense to correct vitamin D deficiencies because sufficient levels promote general health and might assist immune system function, particularly in vulnerable population segments [123]. Scientific evidence confirms that vitamin D supplementation above the suggested levels does not demonstrate enough benefits to treat severe cases of COVID-19 based on trial findings [124,125]. The medical community remains uncertain about how much vitamin D actually helps, while the VIVID trial, along with other extensive studies, aims to find definitive evidence. Health organizations recommend adequate vitamin D intake as a safe secondary measure against COVID-19 while retaining their stance that being free from vitamin D deficiency is a reasonable enough form of protection [42].

## 7. Role of Vitamin D in Long COVID Patients

Vitamin D has been explored as a potential factor in patients experiencing long COVID (post-acute sequelae of SARS-CoV-2 infection), but evidence is still emerging. The immune system reacts to vitamin D because this nutrient both regulates inflammatory responses and controls the production of cytokines. Researchers suppose that vitamin D status determines long COVID development because of sustained inflammation and abnormal immune function [126]. The presence of vitamin D insufficiency directly leads to symptoms of fatigue alongside muscle-related discomfort and weakness, which frequently develop in patients with long COVID [127]. Vitamin D may benefit neurological health by exhibiting anti-inflammatory properties and neuroprotective functions, which affect both mental health symptoms and cognitive function associated with long COVID [128]. The research results from observational studies show that individuals with deficient vitamin D levels have increased disease severity and a lengthened duration of symptoms, like fatigue, muscle weakness, brain fog, and mood disruption, following COVID-19 infection [129]. The Journal of Clinical Endocrinology & Metabolism (2022) conducted a study that connected vitamin D deficiency to worse severity, as well as longer-lasting symptoms of long COVID [130]. The recovery rate for patients with vitamin D levels higher than 30 ng/mL proved more favorable than that for patients demonstrating deficiency [131]. Limited research based on a small number of participants indicates that providing vitamin D supplementation restores long COVID symptoms when patients receive treatment for their vitamin D deficiency [132]. Ongoing research and planned studies continue to evaluate the effects of vitamin D supplements on the treatment of long COVID [133]. Scientific research has not established enough evidence to confirm that vitamin D supplements enhance long COVID outcomes. Health organizations want healthcare professionals to check vitamin D levels in recovering COVID-19 patients who experience ongoing symptoms. The medical community recommends treating identified vitamin D shortcomings because this approach benefits general health and may reduce symptoms [134]. Long COVID patients can receive vitamin D doses of 800–2000 IU per day to achieve safe serum levels above 30 ng/mL in their blood [135,136]. Patients should avoid the self-administration of high doses of vitamin D because they require medical consultation to establish a suitable treatment plan.

## 8. Highlights

- High-dose vitamin D supplementation has not been recommended as a routine treatment for preventing or treating COVID-19 because researchers lack sufficient proof of its effectiveness.
- The relationship between vitamin D deficiency with levels under 20 ng/mL in serum 25(OH)D and extreme COVID-19 outcomes remains under debate regarding any causal connection.
- Patients with long COVID show widespread vitamin D deficiency patterns, which concurrently worsen all their persistent symptoms, including fatigue, muscle weakness, cognitive impairment, and mood disturbances.
- Evidence is insufficient to draw firm conclusions on whether vitamin D deficiency correction through supplementation helps long COVID patients, irrespective of muscle pain symptoms and fatigue, yet research using randomized controlled trials continues to provide insights into this topic.

## 9. Limitations of This Review

Among the major drawbacks of the studies identified and included in this review is the small sample size, which raises questions about the results obtained. Another deficiency is that the studies were conducted in different geographical areas, and therefore, the populations studied were likely affected by different variants of the virus, with the severity of COVID-19 cases being determined by the circulating variant. There is also a lack of a global standard of care, with the basic therapeutic regimen varying from one study to another. Other limitations of the studies reviewed include the lack of data on the prevalence of vitamin D deficiency in the population at baseline. Different vitamin D formulations (cholecalciferol and/or calcitriol) were also used, thus making it impossible to assess the effectiveness of a particular formula. In many cases, the dose of vitamin D was higher than the standard doses dispensed without a medical prescription, making it unlikely that patients would take the recommended doses without medical supervision. Although there is evidence from observational studies that vitamin D plays a role in immune function, interventional trials have had rather disappointing results. One significant problem is that most studies did not select patients according to the degree of vitamin D deficiency, and thus, vitamin D supplementation may not have had a beneficial effect on a person with normal or high levels. The meta-analysis results indicate that vitamin D supplementation does not decrease COVID-19 mortality or the number of ICU hospitalizations or mechanical ventilation procedures, and researchers require well-designed randomized trials with a significant number of patients to properly analyze the potential advantages of vitamin D usage for COVID-19 patients.

## 10. Conclusions

Experts have recognized vitamin D as an essential factor for immune system management that strengthens initial defense systems against pathogens while controlling unnecessary immune responses. Studies on vitamin D in COVID-19 have a biological basis because sufficient vitamin D enhances immune virus clearance and minimizes harmful inflammatory responses leading to severe lung injuries. Experimental observations support vitamin D systems in controlling COVID-19 but studies have yet to demonstrate clear clinical advantages in trials and scientists continue their investigations.

This narrative review highlighted that there are insufficient data to support the use of vitamin D supplementation as a treatment for COVID-19 patients, but individuals who are vitamin D-deficient may benefit from vitamin D supplementation as a nutritional correction for those with deficient diets or for those who have low levels of vitamin D, as determined via biochemical tests. Long COVID patients should preserve adequate vitamin D levels because this strengthens their immune and muscular systems, even if only providing modest recovery improvement. Multiple clinical investigations must take place before medical experts can endorse the use of vitamin D as an exclusive therapeutic approach for patients suffering from long COVID. Scientific studies lack convincing evidence that shows that high-dose vitamin D can effectively be used to treat long COVID, although maintaining adequate vitamin D levels remains safe, has general health benefits, and can possibly aid in mediating numerous long COVID symptoms.

## Figures and Tables

**Table 1:** Clinical studies with focus on vitamin D effects on COVID-19.

Study Title	Registry ID	Phase	Study Type	Recruitment Status	Results Posted	Registry Link
Efficacy of Vitamin D Treatment in Patients Diagnosed with Pneumonia who Require Hospital Admission and have Vitamin D Deficiency and a Positive Diagnosis for SARS-CoV-2 (COVID-19)	EudraCT 2020-001960-28	– (Not stated)	I	Ongoing	No	EU CTR [35]
Preventing Disease Aggravation in COVID-19 by High Dose Vitamin D: a Randomized Trial (COVIT-D)	EudraCT 2020-001793-30	– (Not stated)	I	Prematurely Ended	No	EU CTR [35]
Usefulness of Vitamin D on Morbidity and Mortality of SARS-CoV-2 Infection (COVID-19) at the Central University Hospital of Asturias	EudraCT 2020-002274-28	– (Not stated)	I	Completed	No	EU CTR [35]
COVID-19 and Vitamin D Supplementation: a Multicenter Randomized Controlled Trial of High Dose versus Standard Dose Vitamin _3_ in High-Risk COVID-19 Patients	EudraCT 2020-001602-34	– (Not stated)	I	Completed	No	EU CTR [35]
COVID-19 Prophylaxis with Hydroxychloroquine, Vitamin D, and Zinc Supplementation in Danish Nursing Home Residents—a Randomized Controlled Trial	EudraCT 2020-001363-85	– (Not stated)	I	Prematurely Ended	Yes (Posted)	EU CTR [35]
A Randomized Clinical Trial (IIIb) of Efficacy of a Single Dose of Tocilizumab or a Combination of Tocilizumab plus Vitamin D for the Treatment of COVID-19 Hyperimmune Complications	EudraCT 2020-001903-17	IIIb	I	Ongoing	No	EU CTR [35]
Phase III Randomized Open-Label Trial to Evaluate High-Dose Cholecalciferol (Vitamin D3) in Patients with COVID-19 Pneumonia	EudraCT 2020-002312-43	III	I	Completed	No	EU CTR [35]
COVitaminD Trial: Prevention of Complications from COVID-19 in Cancer Patients Under Active Treatment	EudraCT 2020-002119-23	– (Not stated)	I	Prematurely Ended	No	EU CTR [35]
Multicenter, Double-blind, Randomized Trial to Evaluate the Efficacy of Calcifediol Soft Capsules versus Placebo in Reducing Hospital Admissions in Patients with COVID-19	EudraCT 2021-000316-31	– (Not stated)	I	Prematurely Ended	Yes (Posted)	EU CTR [35]
Prevention and Treatment with Calcifediol of Coronavirus COVID-19–Induced Acute Respiratory Syndrome (COVIDIOL)	EudraCT 2020-001717-20	– (Not stated)	I	Prematurely Ended	No	EU CTR [35]
Prevention and Treatment with Calcifediol of COVID-19–Induced Acute Respiratory Syndrome (COVIDIOL trial, Spain) [24]	NCT04366908	2	I	Recruiting (ongoing)	No	CT.gov [36]
Oral 25-hydroxyvitamin D3 and COVID-19 (Iran)	NCT04386850	2/3	I	Recruiting (ongoing)	No	CT.gov [37]
Evaluation of the Relationship Between Zinc, Vitamin D, and B12 Levels in COVID-19-Positive Pregnant Women (Turkey)	NCT04407572	N/A	O	Completed	No	CT.gov [38,39]
International ALLIANCE Study of Therapies to Prevent Progression of COVID-19 (includes Vitamin D3 arm)	NCT04395768	2	I	Recruiting ongoing	No	CT.gov [40,41]
Vitamin D for COVID-19 Trial (VIVID—U.S.)	NCT04536298	3	I	Completed	No (No results posted)	CT.gov [42]
Effect of Vitamin D on Morbidity and Mortality of the COVID-19 (COVID-VIT-D Trial) (Spain/Argentina)	NCT04552951	3	I	Completed (status last known)	No (No results posted)	CT.gov [43,44]
Prevention of COVID-19 With Oral Vitamin D Supplementation (CORONAVIT trial, UK)	NCT04579640	3	I	Completed	No (No results posted)	CT.gov [22,45]
High-Dose Vitamin D Supplementation in COVID-19 Patients (Angers trial, France)	EudraCT Number: 2020-001602-34	3	I	Completed (status last known)	No	CT.gov [46]
The LEAD COVID-19 Trial: Low-risk, Early Aspirin, and Vitamin D to Reduce COVID-19 Hospitalizations (LEAD COVID-19, USA)	NCT04363840	2	I	Withdrawn (lack of funding)	No	CT.gov [47,48]
COVID-19 and High-dose Vitamin D Supplementation in High-Risk Older Patients (COVIT-TRIAL, Europe)	NCT04344041	3	I	Completed (status last known)	No	CT.gov [27,46,49]
Pilot Study of Vitamin D in COVID-19 Patients (single-arm trial, USA)	NCT04407286	N/A	I (single-arm)	Completed (status last known)	No	CT.gov [50,51]
Trial of Combination Therapy to Treat COVID-19 Infection ProgenaBiome	NCT04482686	1	I	Completed (status last known)	No	CT.gov [52]
Vitamin D Supplementation in Patients With COVID-19 (Brazil)	NCT04449718	3	I	Completed (status last known)	No	CT.gov [53,54,55,56]
High Dose Vitamin-D Substitution in Patients With COVID-19: a Randomized Controlled, Multi-Center Study (VitCov)	NCT04525820	N/A	I	Completed (status last known)	No	CT.gov [57,58]
Efficacy of Vitamin D Supplementation to Prevent the Risk of Acquiring COVID-19 in Healthcare Workers (COVID-19)	NCT04535791	3	I	Completed (status last known)	No	CT.gov [28,59]
Efficacy of Vitamin D Treatment in Pediatric Patients Hospitalized by COVID-19 (Mexico)	NCT04502667	3	I	Completed (status last known)	No	CT.gov [60]
Vitamin D, Magnesium, and B12 in COVID-19 (DMB) (Singapore cohort study)	– (No NCT, local study)	N/A	O	Completed	–	[61]
Hydroxychloroquine, Vitamin C, Vitamin D, and Zinc for COVID-19 Prevention (HELP COVID-19 Trial, USA)	NCT04335084	2	I	Active, not recruiting	No	CT.gov [62]
High-Dose vs. Standard-Dose Vitamin D3 in Patients with COVID-19 (SHADE trial, India)	CTRI/2020/06/026189 (no NCT)	–	I	Completed	Published only	CT.gov [26,63]
Prevention of COVID-19 With Oral Vitamin D Supplemental Therapy in Essential Healthcare Teams (PROTECT)	NCT04483635	3	I	Completed	Published	CT.gov [64,65]
Vitamin D and Zinc Supplementation for Improving Treatment Outcomes Among COVID-19 Patients in India	NCT04641195	3	I	Completed	Published	CT.gov [66,67]
Vitamin D Supplementation in Patients With COVID-19	NCT04449718	N/A	I	Completed	Published	CT.gov [23,53]
Vitamin D and COVID-19 Management (Canada)	NCT04385940	3	I	Completed	No	CT.gov [68]
Investigating the Role of Vitamin D in the Morbidity of COVID-19 Patients (UK)	NCT04386044	N/A	I	Completed	No	CT.gov [69]
Baseline Vitamin D Deficiency and COVID-19 Disease Severity (USA)	NCT04628000	N/A	O	Completed	No	CT.gov [70]
Increased Risk of Severe Coronavirus Disease 2019 in Patients with Vitamin D Deficiency (COVIT-D, Spain)	NCT04403932	N/A	O	Completed	No	CT.gov [71]
Vitamin D Status and Immune-inflammatory Status in Different UK Populations With COVID-19 Infection	NCT04519034	N/A	O	Active, not recruiting	No	CT.gov [72]
Should Ranges of Vitamin D be Redefined to Prevent or Treat Viral Infections? (Turkey)	NCT04394390	N/A	O	Completed	No	CT.gov [73]
Cholecalciferol to Improve the Outcomes of COVID-19 Patients (CARED)	NCT04411446	4	O	Unknown status	No	CT.gov [29,74]
Vitamin D on Prevention and Treatment of COVID-19 (COVITD-19)	NCT04334005	N/A	I	Unknown status	No	CT.gov [75]

I—interventional; O—observational, N/A—non attributed.

**Table 2:** Vitamin D and dosage recommendations.

Population	Recommended Vitamin D Intake	Purpose/Comments	Evidence Level/ Guideline Source
General population	400–800 IU (10–20 µg) daily	Maintain general health and immune support	CDC, WHO, NHS guidelines
At-risk groups (elderly, limited sun exposure, chronic illnesses)	800–2000 IU (20–50 µg) daily	Ensure adequate vitamin D status, potentially reducing severe respiratory infections	NICE (UK), NIH guidelines
Hospitalized COVID-19 patients	No routine high-dose supplementation recommended	Correct vitamin D deficiency if present (typically 1000–2000 IU/day or higher doses under medical supervision)	NIH COVID-19 Treatment Guidelines
COVID-19 prevention (general)	No evidence-based recommendation beyond general intake	Maintaining adequate vitamin D levels might indirectly support immune health	CDC, NIH COVID-19 Treatment Guidelines
Safe upper limit for adults	4000 IU (100 µg) per day	Avoid potential toxicity and hypercalcemia	Institute of Medicine (IOM), NIH guidelines

## Data Availability

Data sharing is not applicable.

## Abbreviations

The following abbreviations are used in this manuscript:

IU	International Unit
ICU	Intensive Care Unit
RCT	Randomized Controlled Trial

## References

[1] Katz J., Yue S., Xue W. (2021). Increased risk for COVID-19 in patients with vitamin D deficiency. Nutrition.

[2] Fakhoury H.M.A., Kvietys P.R., Shakir I., Shams H., Grant W.B., Alkattan K. (2021). Lung-Centric Inflammation of COVID-19: Potential Modulation by Vitamin D. Nutrients.

[3] Karonova T.L., Golovatyuk K.A., Kudryavtsev I.V., Chernikova A.T., Mikhaylova A.A., Aquino A.D., Lagutina D.I., Zaikova E.K., Kalinina O.V., Golovkin A.S. (2022). Effect of Cholecalciferol Supplementation on the Clinical Features and Inflammatory Markers in Hospitalized COVID-19 Patients: A Randomized, Open-Label, Single-Center Study. Nutrients.

[4] Jolliffe D.A., Camargo C.A., Sluyter J.D., Aglipay M., Aloia J.F., Ganmaa D., Bergman P., Bischoff-Ferrari H.A., Borzutzky A., Damsgaard C.T. (2021). Vitamin D supplementation to prevent acute respiratory infections: A systematic review and meta-analysis of aggregate data from randomised controlled trials. Lancet Diabetes Endocrinol..

[5] Takase T., Tsugawa N., Sugiyama T., Ikesue H., Eto M., Hashida T., Tomii K., Muroi N. (2022). Association between 25-hydroxyvitamin D levels and COVID-19 severity. Clin. Nutr. ESPEN.

[6] Akbar M.R., Wibowo A., Pranata R., Setiabudiawan B. (2021). Low Serum 25-hydroxyvitamin D (Vitamin D) Level Is Associated With Susceptibility to COVID-19, Severity, and Mortality: A Systematic Review and Meta-Analysis. Front. Nutr..

[7] Sartini M., Del Puente F., Oliva M., Carbone A., Bobbio N., Schinca E., Giribone L., Cristina M.L. (2024). Preventive Vitamin D Supplementation and Risk for COVID-19 Infection: A Systematic Review and Meta-Analysis. Nutrients.

[8] Raisi-Estabragh Z., Martineau A.R., Curtis E.M., Moon R.J., Darling A., Lanham-New S., Ward K.A., Cooper C., Munroe P.B., Petersen S.E. (2021). Vitamin D and coronavirus disease 2019 (COVID-19): Rapid evidence review. Aging Clin. Exp. Res..

[9] Jeyakumar A., Bhalekar P., Shambharkar P. (2024). Effect of vitamin D supplementation on the immune response to respiratory tract infections and inflammatory conditions: A systematic review and meta-analysis. Hum. Nutr. Metab..

[10] Gibbons J.B., Norton E.C., McCullough J.S., Meltzer D.O., Lavigne J., Fiedler V.C., Gibbons R.D. (2022). Association between vitamin D supplementation and COVID-19 infection and mortality. Sci. Rep..

[11] Vitamin D—Health Professional Fact Sheet. https://ods.od.nih.gov/factsheets/VitaminD-HealthProfessional/.

[12] Hariyanto T.I., Intan D., Hananto J.E., Harapan H., Kurniawan A. (2022). Vitamin D supplementation and COVID-19 outcomes: A systematic review, meta-analysis and meta-regression. Rev. Med. Virol..

[13] Shah K., Varna V.P., Sharma U., Mavalankar D. (2022). Does vitamin D supplementation reduce COVID-19 severity?: A systematic review. Qjm Int. J. Med..

[14] Arroyo-Díaz J.A., Julve J., Vlacho B., Corcoy R., Ponte P., Román E., Navas-Méndez E., Llauradó G., Franch-Nadal J., Domingo P. (2021). Previous Vitamin D Supplementation and Morbidity and Mortality Outcomes in People Hospitalised for COVID19: A Cross-Sectional Study. Front. Public Health.

[15] Martineau A.R., Jolliffe D.A., Hooper R.L., Greenberg L., Aloia J.F., Bergman P., Dubnov-Raz G., Esposito S., Ganmaa D., Ginde A.A. (2017). Vitamin D supplementation to prevent acute respiratory tract infections: Systematic review and meta-analysis of individual participant data. BMJ.

[16] Tomaszewska A., Rustecka A., Lipińska-Opałka A., Piprek R.P., Kloc M., Kalicki B., Kubiak J.Z. (2022). The Role of Vitamin D in COVID-19 and the Impact of Pandemic Restrictions on Vitamin D Blood Content. Front. Pharmacol..

[17] Liu P.T., Stenger S., Li H., Wenzel L., Tan B.H., Krutzik S.R., Ochoa M.T., Schauber J., Wu K., Meinken C. (2006). Toll-like receptor triggering of a vitamin D-mediated human antimicrobial response. Science.

[18] Bishop E.L., Ismailova A., Dimeloe S.K., Hewison M., White J.H. (2020). Vitamin D and Immune Regulation: Antibacterial, Antiviral, Anti-Inflammatory. JBMR Plus.

[19] Jain A., Chaurasia R., Sengar N.S., Singh M., Mahor S., Narain S. (2020). Analysis of vitamin D level among asymptomatic and critically ill COVID-19 patients and its correlation with inflammatory markers. Sci. Rep..

[20] Rhodes J.M., Subramanian S., Laird E., Griffin G., Kenny R.A. (2021). Perspective: Vitamin D deficiency and COVID-19 severity–plausibly linked by latitude, ethnicity, impacts on cytokines, ACE2 and thrombosis. J. Intern. Med..

[21] Grant W.B., Lordan R. (2021). Vitamin D for COVID-19 on Trial: An Update on Prevention and Therapeutic Application. Endocr. Pract..

[22] Jolliffe D.A., Holt H., Greenig M., Talaei M., Perdek N., Pfeffer P., Vivaldi G., Maltby S., Symons J., Barlow N.L. (2022). Effect of a test-and-treat approach to vitamin D supplementation on risk of all cause acute respiratory tract infection and COVID-19: Phase 3 randomised controlled trial (CORONAVIT). BMJ.

[23] Murai I.H., Fernandes A.L., Sales L.P., Pinto A.J., Goessler K.F., Duran C.S.C., Silva C.B.R., Franco A.S., Macedo M.B., Dalmolin H.H.H. (2021). Effect of a Single High Dose of Vitamin D_3_ on Hospital Length of Stay in Patients With Moderate to Severe COVID-19. JAMA.

[24] Entrenas Castillo M.E., Entrenas Costa L.M.E., Vaquero Barrios J.M.V., Alcalá Díaz J.F.A., López Miranda J.L., Bouillon R., Quesada Gomez J.M.Q. (2020). Effect of calcifediol treatment and best available therapy versus best available therapy on intensive care unit admission and mortality among patients hospitalized for COVID-19: A pilot randomized clinical study. J. Steroid Biochem. Mol. Biol..

[25] Lordan R. (2021). Notable Developments for Vitamin D Amid the COVID-19 Pandemic, but Caution Warranted Overall: A Narrative Review. Nutrients.

[26] Rastogi A., Bhansali A., Khare N., Suri V., Yaddanapudi N., Sachdeva N., Puri G.D., Malhotra P. (2022). Short term, high-dose vitamin D supplementation for COVID-19 disease: A randomised, placebo-controlled, study (SHADE study). Postgrad. Med. J..

[27] Annweiler C., Beaudenon M., Gautier J., Gonsard J., Boucher S., Chapelet G., Darsonval A., Fougère B., Guérin O., Houvet M. (2022). High-dose versus standard-dose vitamin D supplementation in older adults with COVID-19 (COVIT-TRIAL): A multicenter, open-label, randomized controlled superiority trial. PLoS Med..

[28] Villasis-Keever M.A., López-Alarcón M.G., Miranda-Novales G., Zurita-Cruz J.N., Barrada-Vázquez A.S., González-Ibarra J., Martínez-Reyes M., Grajales-Muñiz C., Santacruz-Tinoco C.E., Martínez-Miguel B. (2022). Efficacy and Safety of Vitamin D Supplementation to Prevent COVID-19 in Frontline Healthcare Workers. A Randomized Clinical Trial. Arch. Med. Res..

[29] Mariani J., Antonietti L., Tajer C., Ferder L., Inserra F., Cunto M.S., Brosio D., Ross F., Zylberman M., López D.E. (2022). High-dose vitamin D versus placebo to prevent complications in COVID-19 patients: Multicentre randomized controlled clinical trial. PLoS ONE.

[30] Sabico S., Enani M.A., Sheshah E., Aljohani N.J., Aldisi D.A., Alotaibi N.H., Alshingetti N., Alomar S.Y., Alnaami A.M., Amer O.E. (2021). Effects of a 2-Week 5000 IU versus 1000 IU Vitamin D3 Supplementation on Recovery of Symptoms in Patients with Mild to Moderate COVID-19: A Randomized Clinical Trial. Nutrients.

[31] Giustina A., Bilezikian J.P., Adler R.A., Banfi G., Bikle D.D., Binkley N.C., Bollerslev J., Bouillon R., Brandi M.L., Casanueva F.F. (2024). Consensus Statement on Vitamin D Status Assessment and Supplementation: Whys, Whens, and Hows. Endocr. Rev..

[32] Petrelli F., Oldani S., Borgonovo K., Cabiddu M., Dognini G., Ghilardi M., Parati M.C., Petro’ D., Dottorini L., Rea C. (2023). Vitamin D3 and COVID-19 Outcomes: An Umbrella Review of Systematic Reviews and Meta-Analyses. Antioxidants.

[33] Grant W.B., Lahore H., McDonnell S.L., Baggerly C.A., French C.B., Aliano J.L., Bhattoa H.P. (2020). Evidence that Vitamin D Supplementation Could Reduce Risk of Influenza and COVID-19 Infections and Deaths. Nutrients.

[34] Maghbooli Z., Sahraian M.A., Jamalimoghadamsiahkali S., Asadi A., Zarei A., Zendehdel A., Varzandi T., Mohammadnabi S., Alijani N., Karimi M. (2021). Treatment With 25-Hydroxyvitamin D3 (Calcifediol) Is Associated With a Reduction in the Blood Neutrophil-to-Lymphocyte Ratio Marker of Disease Severity in Hospitalized Patients With COVID-19: A Pilot Multicenter, Randomized, Placebo-Controlled, Double-Blinded Clinical Trial. Endocr. Pract..

[35] Clinical Trials Register—Search for COVID-19 and Vitamin D. https://www.clinicaltrialsregister.eu/ctr-search/search?query=covid-19+and+Vitamin+D.

[36] Clinical Trials Register. https://www.clinicaltrialsregister.eu/ctr-search/trial/2020-001717-20/ES.

[37] Study Details | Oral 25-Hydroxyvitamin D3 and COVID-19 | ClinicalTrials.gov. https://clinicaltrials.gov/study/NCT04386850.

[38] Study Details | Evaluation of the Relationship Between Zinc Vitamin D and b12 Levels in the COVID-19 Positive Pregnant Women | ClinicalTrials.gov. https://clinicaltrials.gov/study/NCT04407572.

[39] Bahat P.Y., Talmac M.A., Bestel A., Selcuki N.F.T., Aydın Z., Polat I. (2020). Micronutrients in COVID-19 Positive Pregnancies. Cureus.

[40] Ried K., BinJemain T., Sali A. (2021). Therapies to Prevent Progression of COVID-19, Including Hydroxychloroquine, Azithromycin, Zinc, and Vitamin D3 With or Without Intravenous Vitamin C: An International, Multicenter, Randomized Trial. Cureus.

[41] Study Details | International ALLIANCE Study of Therapies to Prevent Progression of COVID-19 | ClinicalTrials.gov. https://clinicaltrials.gov/study/NCT04395768.

[42] Wang R., DeGruttola V., Lei Q., Mayer K.H., Redline S., Hazra A., Mora S., Willett W.C., Ganmaa D., Manson J.E. (2021). The vitamin D for COVID-19 (VIVID) trial: A pragmatic cluster-randomized design. Contemp. Clin. Trials.

[43] Study Details | Effect of Vitamin D on Morbidity and Mortality of the COVID-19 | ClinicalTrials.gov. https://clinicaltrials.gov/study/NCT04552951.

[44] Cannata-Andía J.B., Díaz-Sottolano A., Fernández P., Palomo-Antequera C., Herrero-Puente P., Mouzo R., Carrillo-López N., Panizo S., Ibañez G.H., Cusumano C.A. (2022). A single-oral bolus of 100,000 IU of cholecalciferol at hospital admission did not improve outcomes in the COVID-19 disease: The COVID-VIT-D—A randomised multicentre international clinical trial. BMC Med..

[45] Trial | NCT04579640. NCT04579640.

[46] Study Details | COVID-19 and Vitamin D Supplementation: A Multicenter Randomized Controlled Trial of High Dose Versus Standard Dose Vitamin D3 in High-Risk COVID-19 Patients (CoVitTrial) | ClinicalTrials.gov. https://clinicaltrials.gov/study/NCT04344041?cond=NCT04344041&rank=1.

[47] Study Details | The LEAD COVID-19 Trial: Low-Risk, Early Aspirin and Vitamin D to Reduce COVID-19 Hospitalizations | ClinicalTrials.gov. https://www.clinicaltrials.gov/study/NCT04363840.

[48] Stroehlein J.K., Wallqvist J., Iannizzi C., Mikolajewska A., Metzendorf M.-I., Benstoem C., Meybohm P., Becker M., Skoetz N., Stegemann M. (2021). Vitamin D supplementation for the treatment of COVID-19: A living systematic review. Cochrane Database Syst. Rev..

[49] Study Details | Vitamin D Supplementation in the Prevention and Mitigation of COVID-19 Infection | ClinicalTrials.gov. https://clinicaltrials.gov/study/NCT04482673?cond=NCT04482673&rank=1.

[50] Study Details | Vitamin D Testing and Treatment for COVID 19 | ClinicalTrials.gov. https://clinicaltrials.gov/study/NCT04407286.

[51] Babajani F., Kakavand A., Mohammadi H., Sharifi A., Zakeri S., Asadi S., Afshar Z.M., Rahimi Z., Sayad B. (2021). COVID-19 and renin angiotensin aldosterone system: Pathogenesis and therapy. Health Sci. Rep..

[52] Study Details | Trial of Combination Therapy to Treat COVID-19 Infection | ClinicalTrials.gov. https://clinicaltrials.gov/study/NCT04482686.

[53] Study Details | Vitamin D Supplementation in Patients with COVID-19 | ClinicalTrials.gov. https://clinicaltrials.gov/study/NCT04449718.

[54] Fernandes A.L., Sales L.P., Santos M.D., Caparbo V.F., Murai I.H., Pereira R.M.R. (2022). Persistent or new symptoms 1 year after a single high dose of vitamin D3 in patients with moderate to severe COVID-19. Front. Nutr..

[55] Fernandes A.L., Murai I.H., Reis B.Z., Sales L.P., Santos M.D., Pinto A.J., Goessler K.F., Duran C.S.C., Silva C.B.R., Franco A.S. (2022). Effect of a single high dose of vitamin D3 on cytokines, chemokines, and growth factor in patients with moderate to severe COVID-19. Am. J. Clin. Nutr..

[56] Murai I.H., Fernandes A.L., Antonangelo L., Gualano B., Pereira R.M.R. (2021). Effect of a Single High-Dose Vitamin D3 on the Length of Hospital Stay of Severely 25-Hydroxyvitamin D-Deficient Patients with COVID-19. Clinics.

[57] Study Details | High Dose Vitamin-D Substitution in Patients with COVID-19: A Randomized Controlled, Multi Center Study | ClinicalTrials.gov. https://www.clinicaltrials.gov/study/NCT04525820.

[58] Jaun F., Boesing M., Lüthi-Corridori G., Abig K., Makhdoomi A., Bloch N., Lins C., Raess A., Grillmayr V., Haas P. (2022). High-dose vitamin D substitution in patients with COVID-19: Study protocol for a randomized, double-blind, placebo-controlled, multi-center study—VitCov Trial. Trials.

[59] Study Details | Efficacy of Vitamin D Supplementation to Prevent the Risk of Acquiring COVID-19 in Healthcare Workers | ClinicalTrials.gov. https://clinicaltrials.gov/study/NCT04535791.

[60] Study Details | Efficacy of Vitamin D Treatment in Pediatric Patients Hospitalized by COVID-19 | ClinicalTrials.gov. https://www.clinicaltrials.gov/study/NCT04502667.

[61] Tan C.W., Ho L.P., Kalimuddin S., Cherng B.P.Z., Teh Y.E., Thien S.Y., Wong H.M., Tern P.J.W., Chandran M., Chay J.W.M. (2020). Cohort study to evaluate the effect of vitamin D, magnesium, and vitamin B12 in combination on progression to severe outcomes in older patients with coronavirus (COVID-19). Nutrition.

[62] Study Details | A Study of Hydroxychloroquine, Vitamin C, Vitamin D, and Zinc for the Prevention of COVID-19 Infection | ClinicalTrials.gov. https://clinicaltrials.gov/study/NCT04335084.

[63] Speakman L., Michienzi S., Badowski M. (2021). Vitamins, supplements and COVID-19: A review of currently available evidence. Drugs Context.

[64] Study Details | Prevention of COVID-19 With Oral Vitamin D Supplemental Therapy in Essential healthCare Teams | ClinicalTrials.gov. https://clinicaltrials.gov/study/NCT04483635.

[65] Ducharme F.M., Tremblay C., Golchi S., Hosseini B., Longo C., White J.H., Coviello D., Quach C., Ste-Marie L.-G., Platt R.W. (2023). Prevention of COVID-19 with oral vitamin D supplemental therapy in essential healthcare teams (PROTECT): Protocol for a multicentre, triple-blind, randomised, placebo-controlled trial. BMJ Open.

[66] Study Details | Vitamin D and Zinc Supplementation for Improving Treatment Outcomes Among COVID-19 Patients in India | ClinicalTrials.gov. https://clinicaltrials.gov/study/NCT04641195.

[67] Sharma K.K., Partap U., Mistry N., Marathe Y., Wang M., Shaikh S., D’Costa P., Gupta G., Bromage S., Hemler E. (2022). Randomised trial to determine the effect of vitamin D and zinc supplementation for improving treatment outcomes among patients with COVID-19 in India: Trial protocol. BMJ Open.

[68] Study Details | Vitamin D and COVID-19 Management | ClinicalTrials.gov. https://www.clinicaltrials.gov/study/NCT04385940.

[69] Study Details | Investigating the Role of Vitamin D in the Morbidity of COVID-19 Patients | ClinicalTrials.gov. https://www.clinicaltrials.gov/study/NCT04386044.

[70] Study Details | Baseline Vitamin D Deficiency and COVID-19 Disease Severity | ClinicalTrials.gov. https://www.clinicaltrials.gov/study/NCT04628000.

[71] Study Details | Increased Risk of Severe Coronavirus Disease 2019 in Patients with Vitamin D Deficiency | ClinicalTrials.gov. https://www.clinicaltrials.gov/study/NCT04403932.

[72] Study Details | Vitamin D Status and Immune-Inflammatory Status in Different UK Populations with COVID-19 Infection | ClinicalTrials.gov. https://www.clinicaltrials.gov/study/NCT04519034.

[73] Study Details | Should Ranges of Vitamin D be Redefined to Prevent or Treat Viral Infections? | ClinicalTrials.gov. https://www.clinicaltrials.gov/study/NCT04394390.

[74] Study Details | Cholecalciferol to Improve the Outcomes of COVID-19 Patients | ClinicalTrials.gov. https://www.clinicaltrials.gov/study/NCT04411446#study-overview.

[75] Study Details | Vitamin D on Prevention and Treatment of COVID-19 | ClinicalTrials.gov. https://clinicaltrials.gov/study/NCT04334005.

[76] Zhang Y., Li J., Yang M., Wang Q. (2023). Effect of vitamin D supplementation on COVID-19 patients: A systematic review and meta-analysis. Front. Nutr..

[77] Grant W.B., Wimalawansa S.J., Pludowski P., Cheng R.Z. (2025). Vitamin D: Evidence-Based Health Benefits and Recommendations for Population Guidelines. Nutrients.

[78] Vitamin D—NHS. https://www.nhs.uk/conditions/vitamins-and-minerals/vitamin-d/.

[79] SACN Vitamin D and Health Report—GOV.UK. https://www.gov.uk/government/publications/sacn-vitamin-d-and-health-report.

[80] Del Valle H.B., Yaktine A.L., Taylor C.L., Ross A.C. (2011). Dietary Reference Intakes for Calcium and Vitamin D.

[81] Dramé M., Cofais C., Hentzien M., Proye E., Coulibaly P.S., Demoustier-Tampère D., Destailleur M.-H., Lotin M., Cantagrit E., Cebille A. (2021). Relation between Vitamin D and COVID-19 in Aged People: A Systematic Review. Nutrients.

[82] COVID-19 Rapid Guideline: Vitamin D | Guidance | NICE. https://www.nice.org.uk/guidance/ng191/chapter/Recommendations-for-research.

[83] COVID-19 Rapid Guideline: Vitamin, D. COVID-19 Rapid Guideline: Vitamin D, December 2020. https://www.ncbi.nlm.nih.gov/books/NBK566063/.

[84] Nitulescu G.M., Paunescu H., Moschos S.A., Petrakis D., Nitulescu G., Ion G.N.D., Spandidos D.A., Nikolouzakis T.K., Drakoulis N., Tsatsakis A. (2020). Comprehensive analysis of drugs to treat SARS-CoV-2 infection: Mechanistic insights into current COVID-19 therapies (Review). Int. J. Mol. Med..

[85] Leaf D.E., Ginde A.A. (2021). Vitamin D_3_ to Treat COVID-19. JAMA.

[86] SACN Rapid Review: Vitamin D and Acute Respiratory Tract Infections—GOV.UK. https://www.gov.uk/government/publications/sacn-rapid-review-vitamin-d-and-acute-respiratory-tract-infections.

[87] De Smet D., De Smet K., Herroelen P., Gryspeerdt S., A Martens G. (2021). Serum 25(OH)D Level on Hospital Admission Associated With COVID-19 Stage and Mortality. Am. J. Clin. Pathol..

[88] Panagiotou G., Tee S.A., Ihsan Y., Athar W., Marchitelli G., Kelly D., Boot C.S., Stock N., Macfarlane J., Martineau A.R. (2020). Low serum 25-hydroxyvitamin D (25[OH]D) levels in patients hospitalised with COVID-19 are associated with greater disease severity: Results of a local audit of practice. Clin. Endocrinol..

[89] Hernández J.L., Nan D., Fernandez-Ayala M., García-Unzueta M., Hernández-Hernández M.A., López-Hoyos M., Muñoz-Cacho P., Olmos J.M., Gutiérrez-Cuadra M., Ruiz-Cubillán J.J. (2021). Vitamin D Status in Hospitalized Patients with SARS-CoV-2 Infection. J. Clin. Endocrinol. Metab..

[90] Amrein K., Schnedl C., Holl A., Riedl R., Christopher K.B., Pachler C., Purkart T.U., Waltensdorfer A., Münch A., Warnkross H. (2014). Effect of high-dose vitamin D3on hospital length of stay in critically ill patients with vitamin D deficiency: The VITdAL-ICU randomized clinical trial. JAMA J. Am. Med. Assoc..

[91] Vogiatzi M.G., Jacobson-Dickman E., DeBoer M.D. (2014). Vitamin D supplementation and risk of toxicity in pediatrics: A review of current literature. J. Clin. Endocrinol. Metab..

[92] Auguste B.L., Avila-Casado C., Bargman J.M. (2019). Use of vitamin D drops leading to kidney failure in a 54-year-old man. Can. Med. Assoc. J..

[93] Galior K., Grebe S., Singh R. (2018). Development of Vitamin D Toxicity from Overcorrection of Vitamin D Deficiency: A Review of Case Reports. Nutrients.

[94] Luo X., Liao Q., Shen Y., Li H., Cheng L. (2021). Vitamin D Deficiency Is Associated with COVID-19 Incidence and Disease Severity in Chinese People [corrected]. J. Nutr..

[95] Wang Z., Joshi A., Leopold K., Jackson S., Christensen S., Nayfeh T., Mohammed K., Creo A., Tebben P., Kumar S. (2022). Association of vitamin D deficiency with COVID-19 infection severity: Systematic review and meta-analysis. Clin. Endocrinol..

[96] Ling S.F., Broad E., Murphy R., Pappachan J.M., Pardesi-Newton S., Kong M.F., Jude E.B. (2020). High-Dose Cholecalciferol Booster Therapy is Associated with a Reduced Risk of Mortality in Patients with COVID-19: A Cross-Sectional Multi-Centre Observational Study. Nutrients.

[97] Pereira M., Dantas Damascena A.D., Galvão Azevedo L.M.G., de Almeida Oliveira T.D.A., da Mota Santana J.D.M. (2022). Vitamin D deficiency aggravates COVID-19: Systematic review and meta-analysis. Crit. Rev. Food Sci. Nutr..

[98] Yisak H., Ewunetei A., Kefale B., Mamuye M., Teshome F., Ambaw B., Yitbarek G.Y. (2021). Effects of Vitamin D on COVID-19 Infection and Prognosis: A Systematic Review. Risk Manag. Health Policy.

[99] Herrera-Quintana L., Gamarra-Morales Y., Vázquez-Lorente H., Molina-López J., Castaño-Pérez J., Machado-Casas J.F., Coca-Zúñiga R., Pérez-Villares J.M., Planells E. (2021). Bad Prognosis in Critical Ill Patients with COVID-19 during Short-Term ICU Stay regarding Vitamin D Levels. Nutrients.

[100] Kompaniyets L., Goodman A.B., Belay B., Freedman D.S., Sucosky M.S., Lange S.J., Gundlapalli A.V., Boehmer T.K., Blanck H.M. (2021). Body Mass Index and Risk for COVID-19–Related Hospitalization, Intensive Care Unit Admission, Invasive Mechanical Ventilation, and Death—United States, March–December 2020. Mmwr-Morb. Mortal. Wkly. Rep..

[101] Brunvoll S.H., Nygaard A.B., Ellingjord-Dale M., Holland P., Istre M.S., Kalleberg K.T., Søraas C.L., Holven K.B., Ulven S.M., Hjartåker A. (2022). Prevention of COVID-19 and other acute respiratory infections with cod liver oil supplementation, a low dose vitamin D supplement: Quadruple blinded, randomised placebo controlled trial. BMJ.

[102] Oristrell J., Oliva J.C., Casado E., Subirana I., Domínguez D., Toloba A., Balado A., Grau M. (2022). Vitamin D supplementation and COVID-19 risk: A population-based, cohort study. J. Endocrinol. Investig..

[103] Romero-Ibarguengoitia M.E., Gutiérrez-González D., Cantú-López C., Sanz-Sánchez M.Á., González-Cantú A. (2023). Effect of Vitamin D_3_ Supplementation vs. Dietary–Hygienic Measures on SARS-CoV-2 Infection Rates in Hospital Workers with 25-Hydroxyvitamin D3 [25(OH)D3] Levels ≥20 ng/mL. Microorganisms.

[104] Ma H., Zhou T., Heianza Y., Qi L. (2021). Habitual use of vitamin D supplements and risk of coronavirus disease 2019 (COVID-19) infection: A prospective study in UK Biobank. Am. J. Clin. Nutr..

[105] Parant F., Bouloy J., Haesebaert J., Bendim’red L., Goldet K., Vanhems P., Henaff L., Gilbert T., Cuerq C., Blond E. (2022). Vitamin D and COVID-19 Severity in Hospitalized Older Patients: Potential Benefit of Prehospital Vitamin D Supplementation. Nutrients.

[106] Cangiano B., Fatti L.M., Danesi L., Gazzano G., Croci M., Vitale G., Gilardini L., Bonadonna S., Chiodini I., Caparello C.F. (2020). Mortality in an Italian nursing home during COVID-19 pandemic: Correlation with gender, age, ADL, vitamin D supplementation, and limitations of the diagnostic tests. Aging.

[107] Oristrell J., Oliva J.C., Subirana I., Casado E., Domínguez D., Toloba A., Aguilera P., Esplugues J., Fafián P., Grau M. (2021). Association of calcitriol supplementation with reduced COVID-19 mortality in patients with chronic kidney disease: A population-based study. Biomedicines.

[108] Cereda E., Bogliolo L., Lobascio F., Barichella M., Zecchinelli A.L., Pezzoli G., Caccialanza R. (2021). Vitamin D supplementation and outcomes in coronavirus disease 2019 (COVID-19) patients from the outbreak area of Lombardy, Italy. Nutrition.

[109] Annweiler G., Corvaisier M., Gautier J., Dubée V., Legrand E., Sacco G., Annweiler C. (2020). Vitamin D Supplementation Associated to Better Survival in Hospitalized Frail Elderly COVID-19 Patients: The GERIA-COVID Quasi-Experimental Study. Nutrients.

[110] Hastie C.E., Mackay D.F., Ho F., Celis-Morales C.A., Katikireddi S.V., Niedzwiedz C.L., Jani B.D., Welsh P., Mair F.S., Gray S.R. (2020). Vitamin D concentrations and COVID-19 infection in UK Biobank. Diabetes Metab. Syndr. Clin. Res. Rev..

[111] Hosseini B., El Abd A., Ducharme F.M. (2022). Effects of Vitamin D Supplementation on COVID-19 Related Outcomes: A Systematic Review and Meta-Analysis. Nutrients.

[112] Kaufman H.W., Niles J.K., Kroll M.H., Bi C., Holick M.F. (2020). SARS-CoV-2 positivity rates associated with circulating 25-hydroxyvitamin D levels. PLoS ONE.

[113] Meltzer D.O., Best T.J., Zhang H., Vokes T., Arora V., Solway J. (2020). Association of Vitamin D Status and Other Clinical Characteristics With COVID-19 Test Results. JAMA Netw. Open.

[114] Smolders J., van den Ouweland J., Geven C., Pickkers P., Kox M. (2021). Letter to the Editor: Vitamin D deficiency in COVID-19: Mixing up cause and consequence. Metabolism.

[115] Merzon E., Tworowski D., Gorohovski A., Vinker S., Cohen A.G., Green I., Frenkel-Morgenstern M. (2020). Low plasma 25(OH) vitamin D level is associated with increased risk of COVID-19 infection: An Israeli population-based study. FEBS J..

[116] Carpagnano G.E., Di Lecce V., Quaranta V.N., Zito A., Buonamico E., Capozza E., Palumbo A., Di Gioia G., Valerio V.N., Resta O. (2021). Vitamin D deficiency as a predictor of poor prognosis in patients with acute respiratory failure due to COVID-19. J. Endocrinol. Investig..

[117] Baktash V., Hosack T., Patel N., Shah S., Kandiah P., Abbeele K.V.D., Mandal A.K.J., Missouris C.G. (2021). Vitamin D status and outcomes for hospitalised older patients with COVID-19. Postgrad. Med. J..

[118] Im J.H., Je Y.S., Baek J., Chung M.-H., Kwon H.Y., Lee J.-S. (2020). Nutritional status of patients with COVID-19. Int. J. Infect. Dis..

[119] Taha R., Abureesh S., Alghamdi S., Hassan R.Y., Cheikh M.M., Bagabir R.A., Almoallim H., Abdulkhaliq A. (2021). The Relationship Between Vitamin D and Infections Including COVID-19: Any Hopes?. Int. J. Gen. Med..

[120] Alcala-Diaz J.F., Limia-Perez L., Gomez-Huelgas R., Martin-Escalante M.D., Cortes-Rodriguez B., Zambrana-Garcia J.L., Entrenas-Castillo M., Perez-Caballero A.I., López-Carmona M.D., Garcia-Alegria J. (2021). Calcifediol Treatment and Hospital Mortality Due to COVID-19: A Cohort Study. Nutrients.

[121] Athanassiou L., Kostoglou-Athanassiou I., Nikolakopoulou S., Konstantinou A., Mascha O., Siarkos E., Samaras C., Athanassiou P., Shoenfeld Y. (2024). Vitamin D Levels as a Marker of Severe SARS-CoV-2 Infection. Life.

[122] Weir E.K., Thenappan T., Bhargava M., Chen Y. (2020). Does vitamin D deficiency increase the severity of COVID-19?. Clin. Med..

[123] Albergamo A., Apprato G., Silvagno F. (2022). The Role of Vitamin D in Supporting Health in the COVID-19 Era. Int. J. Mol. Sci..

[124] Huang L., Song Z., Lu C., Wang S., Guo C., Lai X.-H., Zhao Z. (2024). A narrative review focusing on randomized clinical trials of vitamin D supplementation for COVID-19 disease. Front. Nutr..

[125] Schoenmakers I., Fraser W.D., Forbes A. (2023). Vitamin D and acute and severe illness—A mechanistic and pharmacokinetic perspective. Nutr. Res. Rev..

[126] Atieh O., Daher J., Durieux J.C., Abboud M., Labbato D., Baissary J., Koberssy Z., Ailstock K., Cummings M., Funderburg N.T. (2025). Vitamins K2 and D3 Improve Long COVID, Fungal Translocation, and Inflammation: Randomized Controlled Trial. Nutrients.

[127] Chadda K.R., Roberts S.A., Lugg S.T., Faniyi A.A., Faustini S.E., Webster C., Duffy J.E., Hewison M., Shields A., Richter A.G. (2024). Vitamin D deficiency and duration of COVID-19 symptoms in UK healthcare workers. Front. Med..

[128] Menéndez S.G., Giménez V.M.M., Holick M.F., Barrantes F.J., Manucha W. (2022). COVID-19 and neurological sequelae: Vitamin D as a possible neuroprotective and/or neuroreparative agent. Life Sci..

[129] Hikmet R.G., Wejse C., Agergaard J. (2023). Effect of Vitamin D in Long COVID Patients. Int. J. Environ. Res. Public Health.

[130] di Filippo L., Frara S., Nannipieri F., Cotellessa A., Locatelli M., Querini P.R., Giustina A. (2023). Low Vitamin D Levels Are Associated With Long COVID Syndrome in COVID-19 Survivors. J. Clin. Endocrinol. Metab..

[131] Bilezikian J.P., Formenti A.M., Adler R.A., Binkley N., Bouillon R., Lazaretti-Castro M., Marcocci C., Napoli N., Rizzoli R., Giustina A. (2021). Vitamin D: Dosing, levels, form, and route of administration: Does one approach fit all?. Rev. Endocr. Metab. Disord..

[132] Barrea L., Verde L., Grant W.B., Frias-Toral E., Sarno G., Vetrani C., Ceriani F., Garcia-Velasquez E., Contreras-Briceño J., Savastano S. (2022). Vitamin D: A Role Also in Long COVID-19?. Nutrients.

[133] Hussein A.A.R.M., Galal I., Amin M.T., Moshnib A.A., Makhlouf N.A., Makhlouf H.A., Abd-Elaal H.K., Kholief K.M.S., Tawab D.A.A., Eldin K.A.K. (2022). Prevalence of vitamin D deficiency among patients attending Post COVID-19 follow-up clinic: A cross-sectional study. Eur. Rev. Med. Pharmacol. Sci..

[134] O’donovan J., Cheong J., Chambler D. (2023). Vitamin D Levels in COVID-19 Patients Admitted to Intensive Care. Health.

[135] Sabit H., Abdel-Ghany S., Abdallah M.S., Abul-Maaty O., Khoder A.I., Shoman N.A., Farrag M.S., Martasek P., Noreddin A.M., Nazih M. (2024). Vitamin D: A key player in COVID-19 immunity and lessons from the pandemic to combat immune-evasive variants. Inflammopharmacology.

[136] Griffin G., Hewison M., Hopkin J., Kenny R., Quinton R., Rhodes J., Subramanian S., Thickett D. (2020). Vitamin D and COVID-19: Evidence and recommendations for supplementation. R. Soc. Open Sci..

